# The structural and health policy environment for delivering integrated HIV and substance use disorder treatments in Puerto Rico

**DOI:** 10.1186/s12913-017-2174-7

**Published:** 2017-03-23

**Authors:** Jared A. Leff, Diana Hernández, Paul A. Teixeira, Pedro C. Castellón, Daniel J. Feaster, Allan E. Rodriguez, Jorge L. Santana-Bagur, Sandra Miranda De León, José Vargas Vidot, Lisa R. Metsch, Bruce R. Schackman

**Affiliations:** 1 Department of Healthcare Policy & Research, Weill Cornell Medical College, 425 East 61st Street, Suite 301, New York, NY 10065 USA; 2 Department of Sociomedical Sciences, Mailman School of Public Health, Columbia University, New York, NY USA; 3 Department of Public Health Sciences, University of Miami Miller School of Medicine, Miami, FL USA; 4 Department of Medicine, University of Miami Miller School of Medicine, Miami, FL USA; 5 Department of Medicine, University of Puerto Rico School of Medicine, San Juan Puerto Rico, USA; 6 Puerto Rico Department of Health, San Juan Puerto Rico, USA; 7 Iniciativa Comunitaria de Investigación, San Juan Puerto Rico, USA

**Keywords:** AIDS, HIV, Injection drug use, Mental health, Qualitative analysis

## Abstract

**Background:**

HIV prevalence in Puerto Rico is nearly twice that of the mainland United States, a level that was substantially fueled by injection drug use. Puerto Rico has a longstanding history of health provision by the public sector that directly affects how HIV and substance use disorder (SUD) treatment services are provided and funded. As part of pre-implementation research for a randomized trial of a community-level intervention to enhance HIV care access for substance users in San Juan, Puerto Rico, we sought to understand the structural and health policy environment for providing HIV and SUD treatments.

**Methods:**

We conducted semi-structured qualitative interviews (*n* = 8) with government and program administrators in English and Spanish. Data were analyzed to identify dominant and recurrent themes.

**Results:**

Participants discussed how lack of integration among medical and mental health service providers, lack of public transportation, and turnover in appointed government officials were barriers to integrated HIV and SUD treatment. Federal funding for support services for HIV patients was a facilitator. The Affordable Care Act has limited impact in Puerto Rico because provisions related to health insurance reform do not apply to U.S. territories.

**Discussion and Conclusions:**

Implications for intervention design include the need to provide care coordination for services from multiple providers, who are often physically separated and working in different reimbursement systems, and the potential for mobile and patient transportation services to bridge these gaps. Continuous interaction with political leaders is needed to maintain current facilitators. These findings are relevant as the current economic crisis in Puerto Rico affects funding, and may be relevant for other settings with substance use-driven epidemics.

**Electronic supplementary material:**

The online version of this article (doi:10.1186/s12913-017-2174-7) contains supplementary material, which is available to authorized users.

## Background

HIV prevalence in Puerto Rico is nearly twice that of the mainland United States [1, 2] with injection drug use playing a large role in transmission [1–3]. Studies of injection drug users (IDUs) in Puerto Rico have found a higher frequency of drug use, needle sharing, reduced time to HIV sero-conversion, and greater AIDS-related mortality compared to the U.S. [4, 5].

Puerto Rico has a history of publicly funded healthcare beginning in 1960 when the Puerto Rico government assumed responsibility for the provision of health care services, and began offering universal coverage to residents [6, 7]. However, publicly-funded services became associated with the medically indigent creating a two-tiered system based on ability to pay [8]. Puerto Rico subsequently introduced *La Reforma* (Health Reform) in 1994, resulting in the privatization of public health facilities and contracting with managed care organizations (MCOs). Research on *La Reforma* has been unable to demonstrate an improvement to access or quality of mental health services [6, 7].

*La Reforma* was overhauled and rebranded in 2010 as *Mi Salud* with the goals of integrating physical and behavioral health care and improving access to quality care [8]. Most notably, *Mi Salud* contracted the delivery of mental health services, including most SUD services, to APS Healthcare Puerto Rico (APS-PR) [9]. *Mi Salud* is financed by federal Medicaid allocations to Puerto Rico and Puerto Rico government funds. Future Puerto Rico government funding is uncertain as the local economy and the government’s financial condition worsen [10]. In 2015, Puerto Rico also received $47 million in Ryan White funds and $38.5 million from SAMHSA to finance its care of HIV-infected patients and substance users [11, 12]. Faith-based organizations also play an important role in providing services for substance users in Puerto Rico [13, 14]. These organizations generally provide residential abstinence-only programs with durations as long as 20 months, often located outside of urban centers.

In this article, we explore the structural and health policy environment for providing HIV and SUD treatment services in Puerto Rico. We interviewed key informants representing government and program administrators involved with the allocation and provision of these services in the second half of 2013. Our objective was to describe structural and health policy factors that act as barriers and facilitators of integrated HIV and SUD treatment. We sought to inform hypotheses about how interventions can effectively improve outcomes for HIV-infected substance users and stem the spread of HIV in Puerto Rico and in similar environments with limited resources. Findings are part of the pre-implementation research of a randomized trial of a community-level structural intervention to enhance access to HIV care for substance users in San Juan, Puerto Rico (NCT01792752).

## Methods

### Study setting, participants, and data collection

Participants (*n* = 8) were identified by our clinical trial collaborators in Puerto Rico as the most relevant stakeholders at the time and included: key federal and local government administrators (*n* = 3), HIV and/or SUD treatment program administrators (*n* = 4), and a non-governmental organization administrator (*n* = 1) whose programs were active in the San Juan, PR metro area. The semi-structured interview guide (see Additional file 1) asked about: IDUs and the local HIV epidemic, needs of IDUs, attitudes toward substance use and HIV, mobilization and community engagement, health insurance, and public policy and the political environment. Interviews were conducted by two of the authors (DH and BRS), one of whom is bilingual (DH). One interview was conducted in English, one in Spanish, and the remainder in a mix of English and Spanish.

Interviews were audio recorded and transcribed by a third-party service and appear in the language(s) in which they were conducted. We analyzed interview data for dominant and recurrent themes. Two bilingual authors not involved in the interviews (JAL and PAT) coded the transcripts using an iterative process [15]. Results were reviewed and agreed upon by all members of the study team. Analyses were conducted in NVivo 10 (QSR International, Doncaster, Burlington, MA).

### Ethics approval

This study was reviewed and approved by the Columbia University Medical Center (AAAK8805), Weill Cornell Medicine (1302013624), and University of Puerto Rico (1680213) Institutional Review Boards. Informed consent was obtained from all participants in line with guidelines and procedures from these ethics committees.

## Results

The most salient themes related to the structural and health policy environment for HIV and SUD care in Puerto Rico are presented alongside illustrative quotes. Themes are presented for each of three topics in the interview guide (see Additional file 1): Public Policy and Politics, Health Insurance, and Needs of IDUs.

### Public policy and politics

Several interviewees commented on the influential role of the territory’s Governor in appointing program officials and that top-to-bottom staff turnover is common when a new administration takes office. One government administrator described political turnover in the context of the mainland U.S. as follows:

*[…] let me use New York as an example. When a new administration comes in, the new commissioner is appointed, right, but the deputy commissioner, the senior staff, the directors of the different programs, they are all pretty the same. […] In Puerto Rico, it’s different. When a new administration comes in, the head of the agency is moved out. The deputy is moved out, if there is a deputy. The senior staff are moved out, and the directors of the programs move out.*

This cycling of personnel in-and-out of office creates uncertainty for programs that rely on Puerto Rico government funding and policy making. An HIV treatment program administrator described the need to educate government program officials and to be proactive in order to ensure the continuity of government funding and the uncertainty introduced by the constant change in elected officials.

*[With] new administrations, you get new people that don’t know about HIV. […] So we have to have been teaching, educating people, our bosses, [telling them] ‘Look, it’s done this way. This is done that way. This is not done this way.’*

### Health insurance

All respondents described a lack of integration between mental, behavioral, and physical health care services in Puerto Rico, despite the stated objective of *Mi Salud*, to improve care integration. One treatment program administrator described the situation as:

*20 years ago the government decided to implement access for everybody but… they forget about mental health. Okay, so through the years, they are trying to put mental health [back in] but it’s still, it’s physical health and mental health.*

This artificial barrier between physical and mental health care was frequently cited by program administrators who reported they could not integrate the two because of the structure of the APS-PR contract with *Mi Salud*. Some had tried working with APS-PR to bring mental health staff onsite, but they often encountered resistance and financial burdens. One treatment program administrator reported success in co-locating services, but said implementation was suboptimal:

*The psychologist from APS used the facility of this center. They make the appointments, everything, no communication between the primary care physician and anything. That’s not integration of services. That’s co-location or whatever.*

APS-PR allows for the treatment of opioid-dependence with buprenorphine at a non-APS site, but providers must first obtain additional certification from APS. Reimbursement rates at non-APS sites were described as insufficient, not covering the cost of the service, leading to poor uptake at other sites. A treatment program administrator described some of the consequences of this divided care delivery system.

*We have patients here who are on buprenorphine. [The APS] psychiatrists don’t know our patients, they don’t know anything about HIV, so we had difficulty because a couple of our patients almost died…because a doctor from APS give [a contraindicated medication] to a patient on buprenorphine, and the patient came in and starts having problems.*

Interviewees also described the sources of reimbursement for HIV services. Typically, medical services provided by licensed professionals were described as being reimbursed using *Mi Salud* funds or federal primary care block grant funds, while care coordination or “wrap-around” services were funded by the federal Ryan White program. Another treatment program administrator provided examples on how a community health center providing HIV services may receive funds:

*… I will have my Bureau Primary Health Care 330 grantee dollars [funded by HRSA], so from that pot I will cover the HIV patients’ primary care services. Then I will get [Ryan White] Part C, then I will have the additional support services, some kind of medication. And then I have a contract with the [Ryan White] Part B to get for those patients to be able to access the medications that are not covered under the government health insurance plan [*Mi Salud*] in Puerto Rico that we have not talked about. That is where most of these patients, especially the drug users or the homeless, will be covered under, that is the government health insurance plan.*

A government administrator described a situation where Ryan White funds were used to support the salary of health psychologists to work with patients on medication adherence.

*Todo paciente, toda persona VIH tiene que recibir un* screening *de* mental health *y* substance abuse*. Toda, no importa su situación. Ellos son parte de la clínica, donde, por ejemplo, ellos van a trabajar con la adherencia del paciente a tratamiento. Van a estar pendientes al CD4, a la carga viral. Identificar cualquier situación que dificulte que el paciente sea adherente al tratamiento. El, el propósito o el objetivo, o el foco de su intervención, es asegurar que el paciente VIH se mantenga adherente al tratamiento.*

*[All patients, everyone with HIV, must receive a mental health and substance abuse screening. It doesn’t matter the situation. They (the clients) are part of a clinic where, for example, they are going to work on treatment adherence. They are going to be monitoring the CD4, viral load, to identify whatever factor may be making it difficult to adhere to treatment. The purpose or the objective, or the focus of the intervention is to make sure HIV patients maintain adherence to the treatment.]*

The use of Ryan White funds for screening and brief intervention was justified as being related to HIV treatment (and not solely mental health) and avoids making a referral for the patient to an off-site APS-PR-funded site for these services. However, if other mental health services or SUD treatment services are required, the patient must then go to an APS-PR provider.

In December 2013, when these interviews were conducted, most interviewees spoke of the Governor’s last minute decision to use expected ACA funds to expand Medicaid rather than create a health exchange. One treatment program administrator expressed doubt about the effect of ACA on Medicaid expansion:

*Well, the numbers are around 400,000 are the people in Puerto Rico that doesn’t have any insurance. And with the Health Reform [ACA], they supposed to have the ability to have around 20,000 more people into the Medicaid program.*

This interviewee indicated that a small number of uninsured individuals would have become eligible for insurance under the ACA, and the overwhelming majority of uninsured Puerto Ricans would remain without health insurance, based on the funds expected at that time to be received from ACA. Subsequently, in July 2014 the U.S. Department of Health and Human Services determined that ACA provisions related to health insurance reform do not apply to territories such as Puerto Rico [16]. As a result, $925 million in federal ACA-related grant funds to Puerto Rico for Medicaid expansion were eliminated, although other increases in federal Medicaid funding for Puerto Rico that began in 2011 remained in place [17].

### Needs of IDUs

Many interviewees also discussed the lack of public transportation as a barrier to accessing services and described transportation as an unmet need. One government administrator noted:

*Well,* Mi Salud *does not cover… medical transportation that they [patients] need to get to their appointments, to go to the pharmacy to buy a medication, they don’t have those [services]…*

A treatment program administrator recalled a patient who had travelled a long distance for his mental health appointment. After securing transportation from a friend to get to the clinic, he arrived only to find the provider hadn’t made it to clinic that day.

*… and what happened when (he arrived,) they said to him, ‘Come tomorrow, maybe then,’ (and) he decide, ‘Okay, I won’t go. I won’t go.’*

Transportation remains a significant barrier for the majority of patients due to poor public transportation infrastructure and alternate arrangements must be sought from friends/relatives or programs offering this service.

## Discussion and Conclusions

All of the interviewees described significant barriers to the provision of high-quality integrated care for Puerto Ricans living with HIV and SUD. While Puerto Rico’s *Mi Salud* provides coverage to nearly half of the island’s population, physical and mental health service delivery remains siloed and services are rarely co-located leading to sub-optimal delivery of appropriate services. Lack of public transportation further limits access, especially because of the lack of co-located services. Overall, interviewees described a system where the responsibility for accessing and coordinating physical and mental health services falls primarily on the patient and the patient’s support system.

Wrap-around services for HIV-infected patients that facilitate integrated care funded by the Ryan White Care Act are available in Puerto Rico, but these services are vulnerable to overall cuts in Ryan White funding. Ongoing education of political leadership in Puerto Rico on integrated care needs for HIV and SUD is necessary because of the frequent staff turnover in government agencies. Furthermore, the ACA will have little impact in Puerto Rico because as a territory it is not eligible to receive ACA grant funds.

Our findings, which echo results of previous studies [18, 19], have several implications for the design of interventions to improve treatment for HIV-infected substance users in Puerto Rico. First, interventions should provide care coordination services that assist patients in accessing care from multiple providers who are not co-located and work in different care systems. Second, lack of public transportation is a barrier to care which can be addressed by providing patient transportation services or by bringing care services to patients using mobile vans [20–22]. Third, continuous interaction with political leaders in Puerto Rico is needed to educate them on the value of and resources needed to provide integrated HIV and SUD care.

Our findings illustrate the need for interventions with substance users in Puerto Rico. Substance use and comorbid mental health issues are associated with lower rates of engagement in care and treatment adherence [21, 23, 24]. It is now well established that having a detectable HIV viral load is related to higher rates of transmissions [25, 26]. Inadequate coordination of care in areas with substance use-driven HIV epidemics will perpetuate high rates of HIV incidence.

This study has several limitations. First, qualitative data are subject to researcher bias which we sought to address by having the interviews conducted by researchers with extensive qualitative research experience. Second, the HIV and SUD treatment providers we interviewed were all located in the San Juan, Puerto Rico metropolitan area, and the generalizability of their experience to providers located outside of this area is unknown. Third, we focused on the structural and health policy themes that emerged from our analysis, and did not include content that was more related to clinical management issues and patient attitudes. Finally, while some interviewees noted the overall negative impact of the economic situation in Puerto Rico, and the impact of municipal budget cuts was discussed, our interviews were conducted before the most recent financial crisis in Puerto Rico. These more recent events will likely adversely affect funding and availability of health services in Puerto Rico and may accelerate the departure of health professionals from Puerto Rico [27]. These concerns further emphasize the need to continue to educate local government officials, as well as raising questions for further study about the effect of the financial crisis on the demand and capacity for health services in Puerto Rico, and whether or not greater integration of services has the potential to create savings for the health care system as funding is reduced.

Communities in the U.S. with limited resources and substance use-driven epidemics face similar barriers, particularly rural areas with increased IDU among young adults at high risk for HIV and HCV infections [28–31]. Many of these communities are also facing weak economic environments and may be in states that have decided not to use ACA funds to expand Medicaid coverage. Interventions developed and tested in Puerto Rico could potentially be adapted to these communities. However, Puerto Rico faces some unique challenges due to the current design of the *Mi Salud* health system. These challenges will be addressed in planned future implementation research to investigate the budgetary impact and sustainability in Puerto Rico of the interventions that are being tested in the ongoing clinical trial.

## Additional file

Additional file 1: The open-ended interview guide used during data collection is available as a supplemental file entitled Interview Guide_Community Members_v1.0_ENG.doc. (DOC 54 kb)

## Abbreviations

ACA: Patient Protection and Affordable Care Act
APS-PR: APS Healthcare Puerto Rico
IDU: Injection drug user
MCO: Managed care organization
PR: Puerto Rico
SUD: Substance use disorder
