# Supplementary material for: The structural and health policy environment for delivering integrated HIV and substance use disorder treatments in Puerto Rico
Source: BMC Health Serv Res. 2017 Mar 23;17:232. doi: 10.1186/s12913-017-2174-7 (PMC5364616; doi:10.1186/s12913-017-2174-7)
Supplement: Additional file 1: — The open-ended interview guide used during data collection is available as a supplemental file entitled Interview Guide_Community Members_v1.0_ENG.doc. (DOC 54 kb) [file 12913_2017_2174_MOESM1_ESM.doc]

**DRUG USE & HIV/AIDS IN THE SAN JUAN METRO AREA**

**Key Informant/Community Member Interview Guide**

***Injection Drug Use and the Local HIV Epidemic***

1. Please describe the HIV epidemic among injection drug users (IDUs) in Puerto Rico (in your specific area)? What does the epidemiology data in your jurisdiction reflect about IDUs?
2. What are some common characteristics of IDUs in your area – demographics - age, gender, birthplace, connections to the US, housing status, work or other means of income, family ties, ties to health care, criminal justice, duration of drug use, drug type of preference and of frequent use, needle sharing and other risk behavior. How stable/mobile is the local IDU population? Can we count on the “usuarios” to be in the same general area (save for interactions with the criminal justice system or health care) over an extended period of time- say for the duration of the study period? If not, how do the patterns of migration flow?
3. As far as geography - what are the top 5 areas in the San Juan metro area that you would describe as “hot spots” for IDUs? What are the usual places that IDUs in the community meet to cop and do drugs in the area?
4. What are the prevalent issues and/or specific problems that put IDUs in your area at elevated risk for HIV?

Probe: Are any of the following issues prevalent--- co-morbidities mental health, alcohol and substance abuse, needle sharing, sexual risk behavior, gender nuances, histories of incarceration, violence (interpersonal and structural), etc. ---among IDUs in your area? Please explain.

1. How well do IDUs in your community understand the basics of HIV/AIDS? What could be done to increase understanding?
2. How important do you think HIV/AIDS is compared to other problems people face in your community? What are the more pressing issues facing IDUs?
3. What services are available to IDUs in your area as far as HIV testing? HIV care and treatment options? Drug treatment services? How do IDUs in your community learn about available services for persons living with HIV/AIDS?
4. This study entails access to HIV care delivered in a mobile unit. Do you foresee any difficulty getting this initiative launched? If so, what might those barriers be? Any foreseeable strengths of this approach?
5. In the broader scheme of services, what alternatives do IDUs have for linkage to care? How will the mobile unit measure up against those options?

***Needs of IDUs***

1. Name the top three needs, in your opinion, for IDUs living with HIV/AIDS in your area.
2. What strategies are currently used for HIV prevention among IDUs in your area? Have you considered other ideas to keep IDUs who are negative from contracting HIV?
3. What are the key institutional factors that act as barriers to IDUs from accessing care and using various HIV/AIDS and drug treatment interventions?
4. Tell me how important the following factors are in addressing IDU and HIV/AIDS:
   1. Family issues
   2. Economic pressures, work and the need for survival
   3. Mental health issues
   4. Peers/Friends
   5. History of migration to the US
   6. Housing instability and homelessness
   7. Internal and/or community-level HIV, drug use stigma and homophobia
   8. The role of the church (catholic and evangelical)
   9. Cultural factors including Machismo
   10. others

Probe: Do local social service agencies and care and treatment facilities where you get services for HIV/AIDS provide information and support in these areas? If so, please describe.

1. Which programs that you are aware of target/support IDUs?

***Attitudes toward drug use and HIV***

1. How does the broader community deal with drug use?

Probe: How do you think this impacts HIV/AIDS prevalence and risk behavior among IDUs?

1. What are some of the things that help IDUs engage in safer drug use practices? What are some of the things that make it hard for IDUs to engage in safer drug use practices?
2. What is the attitude toward HIV in your community?

***Mobilization and Community Engagement***

1. What needs to happen in order for basic HIV/AIDS knowledge to translate into behavior change among IDUs?

Probe: What would it take for more IDUs to acknowledge their role to better protect themselves?

Probe: Where do IDUs obtain their knowledge and updated information on HIV/AIDS?

1. How familiar are you with the PR health department’s HIV/AIDS outreach activities for IDUs?

Or

How familiar are you with the outreach activities on the part of local community-based organizations around HIV/AIDS that are targeted to IDUs?

1. How would you describe their efforts (including targeted funding) in reaching out and engaging IDUs in your area who are at risk for HIV? Facilitating linkage to care? Referring to drug treatment?
2. Has the health department or other agency sponsored any special events, educational campaigns or forums to address HIV prevention for IDUs in your area? If so, describe. Have they collaborated in interagency initiatives to target IDUs? If so, what is the nature of collaborations with other key institutions such as corrections, education, religious organizations, etc.?
3. In comparison to five years ago, today’s HIV/AIDS epidemic among IDUs in your local community:
   1. is a more urgent problem
   2. is a less urgent problem
   3. is about the same
   4. is relatively low and has never been a problem

***Health Insurance***

- - - 1. Please describe the process in which HIV patients are enrolled (and re-enrolled) in
         1. Ryan White;
         2. La Reforma;
         3. Medicare;
         4. private insurance;
         5. other insurance?
      2. What criteria do patients have to meet in order to become enrolled and to stay enrolled? Where are they most likely to get relevant enrollment information and be enrolled?
      3. Please explain the reimbursement process for which these payers reimburse for the following health and substance abuse treatment services:
         1. HIV related: 1) HIV tests; 2) HIV clinical visits; 3) HIV medications; 4) HIV labs;
         2. non-HIV medications;
         3. care coordination/social worker service;
         4. outreach;
         5. Substance use-related: 1) detox; 2) methadone treatment; 3) buprenorphine/naloxone treatment; 4) other drug treatment programs?
      4. Does reimbursement occur through billing for individual services or as another form of payment (annual grant to the agency, “bundled payment” for care of a population, etc.)?
      5. Which services are actually billed for in practice? Of the services billed for, how often are they actually reimbursed? How does the reimbursement level for these services compare to what you believe it actually costs to provide the service?
      6. How do you think the Affordable Care Act (ACA) will affect enrollment and reimbursement for HIV medical services, non-HIV medical services, and drug treatment services here in PR? How prepared are health and social service providers to deal with the impending changes?
      7. How do you think the AIDS Drug Assistance Program (ADAP) and other Ryan White programs will change here in PR in the future?

***Public Policy and Politics***

1. What are the most important political issues for IDUs in PR today?
2. How does the local political landscape impact you and your response to HIV among IDUs?

***Action Steps/Moving Forward***

1. What additional partnering agencies/leaders would you bring to the table?
2. In light of all the discussions shared today, what advice would you share regarding the implementation of our project?
3. Is there anything that we didn't cover that you feel is important for me to know?
